# Combinatorial effects of antibiotics and enzymes against dual-species *Staphylococcus aureus* and *Pseudomonas aeruginosa* biofilms in the wound-like medium

**DOI:** 10.1371/journal.pone.0235093

**Published:** 2020-06-25

**Authors:** Rima Fanaei Pirlar, Mohammad Emaneini, Reza Beigverdi, Maryam Banar, Willem B. van Leeuwen, Fereshteh Jabalameli

**Affiliations:** 1 Department of Microbiology, School of Medicine, Tehran University of Medical Sciences, Tehran, Iran; 2 Leiden Center for Applied Bioscience, University of Applied Sciences Leiden, Leiden, The Netherlands; University of Hong Kong, HONG KONG

## Abstract

Bacterial biofilms are one of the major issues in the treatment of chronic infections such as chronic wounds, where biofilms are typically polymicrobial. The synergy between species can occur during most polymicrobial infections, where antimicrobial resistance enhances as a result. Furthermore, self-produced extracellular polymeric substance (EPS) in biofilms results in a high tolerance to antibiotics that complicates wound healing. Since most antibiotics fail to remove biofilms in chronic infections, new therapeutic modalities may be required. Disruption of EPS is one of the effective approaches for biofilm eradication. Therefore, degradation of EPS using enzymes may result in improved chronic wounds healing. In the current study, we investigated the efficacy of trypsin, β-glucosidase, and DNase I enzymes on the degradation of dual-species biofilms of *Pseudomonas aeruginosa* and *Staphylococcus aureus* in a wound-like medium. These species are the two most common bacteria associated with biofilm formation in chronic wounds. Moreover, the reduction of minimum biofilm eradication concentration (MBEC) of meropenem and amikacin was evaluated when combined with enzymes. The minimum effective concentrations of trypsin, β-glucosidase, and DNase I enzymes to degrade biofilms were 1 μg/ml, 8 U/ml, and 150 U/ml, respectively. Combination of 0.15 μg/ml trypsin and 50 U/ml DNase I had a significant effect on *S*. *aureus-P*. *aeruginosa* biofilms which resulted in the dispersal and dissolution of all biofilms. In the presence of the enzymatic mixture, MBECs of antibiotics showed a significant decrease (*p <* 0.05), at least 2.5 fold. We found that trypsin**/**DNase I mixture can be used as an anti-biofilm agent against dual-species biofilms of *S*. *aureus-P*. *aeruginosa*.

## Introduction

One main reason for the healing failure of chronic wounds, which include burn, pressure, diabetic, venous, and arterial ulcers, is the formation of bacterial biofilms. Biofilms are involved in 60–80% of chronic wound infections and are typically multi-species [1–4]. The protective effects of biofilms are enhanced synergistically in multi-species mode [5, 6]. Microorganisms in biofilms are protected against antimicrobials by the self-synthesized extracellular polymeric substance (EPS) holding the bacterial cells together. The EPS also increases resistance to the immune system compared to free-living cells [7]. These protective features of EPS complicate the treatment of biofilm-associated chronic wound infections and are responsible for an enhancement of the effective concentrations of antimicrobial agents in biofilm [8]. Hence, anti-biofilm agents that degrade the matrix and expose biofilm bacteria to the environment can make bacteria more susceptible to the host immune system and antibiotics/antimicrobials [9]. Therefore, the use of novel therapies that specifically disrupt biofilms within chronic wounds is a promising strategy for wound-care therapies. In this context, matrix-degrading enzymes have received particular attention and been used for the degradation of biofilm EPS [10–15]. However, the efficiency of each EPS-degrading enzyme will depend on the EPS composition [16, 17]. Due to the heterogeneity of the EPS, different classes of enzymes or a mixture of enzyme activities may be used for a sufficient degradation of bacterial biofilms [18, 19].

Proteins, polysaccharides, and extracellular DNA (eDNA) are main structural components of the EPS. Proteins pose a substantial component in the biofilm EPS, which are crucial for the maintenance and stability of the biofilm matrix [20–23]. Polysaccharides are major EPS constituents in most biofilms that provide many crucial functions for the biofilms [24, 25]. Another contributing component of bacterial biofilms is eDNA that can be vital for the biofilm by working as a structural scaffold within the EPS matrix [26, 27]. Accordingly, three enzymes, including trypsin, β-glucosidase, and DNase I from different classes of enzymes that target the main components in biofilm EPS were included in this study. β-glucosidase, a glycosidase enzyme, is capable of degrading polysaccharides that have β-1→3 and β-1→4 links between their glucose monomers. These bonds are present in Psl and Pel polysaccharides of *P*. *aeruginosa* biofilm matrix. It was assumed that β-glucosidase could destroy Psl and Pel polysaccharides. Psl, Pel, and alginate are three main polysaccharides in the biofilm matrix of *P*. *aeruginosa*, which are products of *pslD*, *pelF*, and *algD* genes, respectively [28].

DNase I as a nuclease can degrade eDNA of the EPS in different bacterial biofilms. Trypsin is a serine endoprotease that cleaves proteins or peptides and may depolymerize protein contents of bacterial biofilms. It has previously been shown that these two enzymes could disrupt *in vitro* mono-species biofilms of *P*. *aeruginosa*, *S*. *aureus*, *Streptococcus pneumonia*, and *Staphylococcus epidermidis* [10, 15, 16, 29, 30].

*S*. *aureus* and *P*. *aeruginosa* are the two most common etiological agents of chronic wound infections and are both frequently found together in polymicrobial, biofilm-related infections [31].

This study aimed to test certain biofilm-degrading enzymes including trypsin, β-glucosidase, and DNase I for their efficacy in degrading EPS produced within dual-species *S*. *aureus-P*. *aeruginosa* biofilms. Moreover, their effect on the reduction of minimum biofilm eradication concentration (MBEC) of meropenem and amikacin was determined. In this study, the Lubbock chronic wound biofilm model (LCWBM), an *in vitro* model system mimicking the conditions observed in a biofilm infected chronic wound was exploited to carry out the treatment procedure [32, 33].

## Material and methods

### Bacterial strains and culture conditions

Six clinical strains (five *P*. *aeruginosa* and one *S*. *aureus;* listed in Table 1) isolated from patients with infected burn wounds were included in this study. Bacterial isolates were provided by tertiary hospital laboratory in Tehran, Iran, that is affiliated with Tehran University of Medical Sciences. *P*. *aeruginosa* ATCC 27853 and *S*. *aureus* ATCC 29213 were used as standard strains in all experiments. Genotypes of clinical *P*. *aeruginosa* strains based on genes encoding biofilm exopolysaccharides (*algD*, *pelF*, *pslB*, *and pslD*) were previously determined by our colleagues, using PCR method [10]. The most prevalent *S*. *aureus* strain based on drutype (drutype 10di) isolated from burn patients was involved in the study [34]. The bacteria were cultured on tryptic soy agar (TSA) (Gibco, USA) and incubated at 37° C for 24 h to prepare working cultures. All the isolates were stored at -80° C in tryptic soy broth (TSB) with 15% glycerol.

**Table 1:** Overview of strains used in this study.

Species	Strain	Genotype
*Pseudomonas aeruginosa*	ATCC 27853 (PA_0_)	*-*
	PA 1185 (PA_1_)	*pelF*^*+*^, *algD*^*+*^, *pslB*^*-*^, *pslD*^*-*^
	PA 1179 (PA_2_)	*pelF*^*+*^, *algD*^*+*^, *pslB*^*+*^, *pslD*^*+*^
	PA 1162 (PA_3_)	*pelF*^*-*^, *algD*^*+*^, *pslB*^*+*^, *pslD*^*+*^
	PA 1326 (PA_4_)	*pelF*^*-*^, *algD*^*-*^, *pslB*^*-*^, *pslD*^*-*^
	PA 1329 (PA_5_)	*pelF*^*-*^, *algD*^*+*^, *pslB*^*-*^, *pslD*^*-*^
*Staphylococcus aureus*	ATCC 29213 (SA_0_)	-
	SA 639 (SA_1_)	dt 10di

* *pelF*, *algD*, *pslB*, *pslD* are main genes encoding biofilm exopolysaccharides in *P*. *aeruginosa*

### In vitro dual-species biofilm formation

The previously described Lubbock chronic wound biofilm model was used with slight modifications to dual-species biofilm formation [32]. Briefly, 200 μl of the wound-like medium (WLM) containing heparinized human plasma, 45% Bolton broth base (Conda, Spain), 1% gelatin (Merck, Germany), and 5% laked sheep red blood cells, were aseptically introduced in 1.5 ml microtubes. Suspensions containing 1×10^6^ CFU/ml of each strain from the two bacterial species were prepared and then mixed. The mixtures contained following strains: PA_0_-SA_0_, PA_1_-SA_1_, PA_2_-SA_1_, PA_3_-SA_1_, PA_4_-SA_1_, and PA_5_-SA_1_. Subsequently, 10 μl of the mixture with a density of 1×10^6^ CFU/ml was inoculated into microtubes and were then incubated at 37° C for 24 h.

The coagulated samples were examined for biofilm formation by visual inspection using a scanning electron microscope (SEM). Biofilms were rinsed thrice by adding 500 μl sterile normal saline and vortexed for 30s to remove any planktonic cells. After being fixed with 2.5% glutaraldehyde for 1 h at room temperature, samples were dehydrated with serially increasing concentrations of ethanol (50%, 70%, 80%, 90%, and 100%) for 10 minutes at 4° C. For further dehydration, samples were treated once more with 100% ethanol for 30 minutes and then transferred to hexamethyldisilazane (HMDS) for drying. Images were viewed and photographed using a Hitachi S-4160 SEM [32, 35].

### Enzymatic treatments on *S*. *aureus-P*. *aeruginosa* dual-species biofilms

The enzymes used in this study include trypsin (Sigma-Aldrich, St. Louis, USA, 1 μg/vial, ≥ 10,000 BAEE U/mg), β-glucosidase from *Almond* (Sigma, 10–30 U/mg solid), and DNase I from bovine pancreas (Sigma, 2000 Kunitz U/vial, > 2500 U/mg). All enzymes were purchased from Sigma Aldrich (St Louis, MO, USA).

Stock solutions and working dilutions of enzymatic preparations were made with suitable buffers, i.e., sodium acetate buffer pH 5 (β-glucosidase), DNase I reaction buffer pH 7.5, and trypsin reaction buffer pH 8. All buffers were provided by Sigma Company along with the enzyme kit. After the establishment of *S*. *aureus-P*. *aeruginosa* biofilms, 500 μl of sterile normal saline was added and vortexed thrice for 30s to collect the planktonic cells and biofilms separately. The supernatant was removed and biofilms were exposed to the various enzymes prepared at different concentrations (Trypsin: 0.5, 0.75, 1, 1.5 μg/ml, β-glucosidase: 4, 8, 16 U/ml and DNase I: 100, 150, 200 U/ml) for 4, 8 and 18 h at 37° C. The minimum effective concentration and the best contact duration time for each enzyme was determined by testing different concentrations of each enzyme on two biofilm groups (S1 File). All enzyme treatments of biofilm were carried out for 18 h at 37° C with the minimum effective enzyme concentrations (Trypsin: 1 μg/ml, β-glucosidase: 8 U/ml DNase I: 150 U/ml). Following incubation, the colony-forming unit (CFU) in the supernatant were enumerated, and counts were compared to those of non-treated/buffer-treated controls. CFUs were counted after overnight incubation at 37° C. To count the remaining cells in biofilms, after removing supernatant and washing twice with sterile saline solution, samples were sonicated for 45s (NEXTGEN-CB17-LAB750, 40% Amplitude, 0.5 Cycle) and colony count was done as described. Staphylococcus/pseudomonas isolation agar (Sigma, Germany) was used for the plate counting.

All three enzymes were mixed in combinations of two or three, to examine the combinatorial effect of the enzymes on biofilms. After preliminary tests, the effect of DNase I**/**trypsin combination on *S*. *aureus-P*. *aeruginosa* biofilms was analyzed. Trypsin and DNase I were mixed at different lower levels than their minimum effective concentrations, which were previously evaluated (Table 2). All experiments were performed three times in triplicate.

**Table 2:** Enzyme concentration in the mixture.

Trypsin) μg/ml)	DNase I (U/ml(
0.15	30
	50
0.25	50
0.5	
0.5	75

### Bactericidal effect of enzymes on planktonic cells

The bactericidal effect of enzymes on planktonic cells of each strain from each of *S*. *aureus* and *P*. *aeruginosa* species was independently evaluated as described previously [10]. The experiment was done using the minimum effective concentration of each enzyme and lower concentrations (Trypsin: ≤ 1 μg/ml, DNase I: ≤ 150 U/ml, β-glucosidase: ≤ 8 U/ml).

Briefly, 50 μl of Mueller Hinton broth (Merck, Germany) was added to each microtiter plate well (Tissue culture plate 96 wells, SPL, Korea). The enzyme was loaded to each well. Finally, 50 μl of bacterial suspension with a final inoculum of 10^6^ CFU/ml was added to each well. The microtiter plate was then incubated for 20 h at 37° C. Plates were inspected based on bacterial growth. The lowest enzyme concentration that visibly inhibited microbial growth was defined as the minimum inhibitory concentration (MIC) [36]. The minimum bactericidal concentration (MBC) was determined by pipetting 10 μl of each well with a clear suspension onto a TSA. After incubation at 37° C for 24 h, the plates were inspected for the presence of colonies. Inoculated MHB without enzyme and MHB plus enzyme with no bacteria were considered as control groups.

### MIC and MBC determination of antibiotics against bacterial monocultures

Susceptibility of all 8 tested isolates to amikacin and meropenem (Jaber Ebne Hayyan Co, Iran) was determined by the broth microdilution method (MIC range, 0.25 to 512 μg/ml) as recommended by the Clinical and Laboratory Standards Institute (CLSI) [36]. Briefly, 50 μl of cation adjusted Mueller Hinton broth (Merck, Germany) was introduced to each microtiter plate well. The antibiotic at prepared concentrations was loaded to each well. Finally, 50 μl of bacterial suspension with a final inoculum of 10^6^ CFU/ml was added to each well of 96-well microtiter plate and the plate was incubated for 20 h at 37° C. Plates were checked based on bacterial growth. *P*. *aeruginosa* ATCC 27853 and *S*. *aureus* ATCC 29213 were used as quality control strains for susceptibility testing. To determine the MBC, 10 μl of the suspension from clear wells with no visible growth was plated on TSA, in triplicate. Following the overnight incubation at 37° C, the growth on TSA was checked. The lowest concentration of the antibiotic that made a 99.9% CFUs reduction of the initial inoculum of planktonic culture was recorded as MBC. In this study, amikacin and meropenem were tested because they are part of the treatment regimen used for burn patients in the studied hospital.

### MBC measurement of antibiotics against planktonic co-cultures of susceptible isolates

Equal amounts of both bacteria containing 10^6^ CFU/ml were inoculated into 200 μl WLM to determine the MBCs of meropenem and amikacin. Bacterial suspensions were grown at 37° C with vigorous shaking to prevent the coagulation of the medium for 24 h. After incubation, various concentrations of antibiotic in WLM from 10 through 1000 μg/ml were added directly to the microtubes. After reincubation at 37° C for 20 h on a shaking device, 10 μl of the antibiotic-treated culture was plated onto a staphylococcus/pseudomonas isolation agar and incubated overnight at 37° C. The lowest antibiotic concentration that resulted in a 99.9% reduction of CFUs with respect to that of the control growth was interpreted as MBC for *S*. *aureus*, or *P*. *aeruginosa* isolates in planktonic co-cultures. Inoculated WLM without antibiotic was used as negative control [32, 37].

### MBEC determination of antibiotics against dual-species biofilms of susceptible isolates

MBEC assay for *S*. *aureus-P*. *aeruginosa* biofilms was performed as follows: the biofilms (SA_1_-PA_1_, SA_1_-PA_2_, and SA_0_-PA_0_) were washed with sterile phosphate buffered saline (PBS) to remove non-adherent cells. Then 200 μl of particular antibiotic dilution in WLM with final concentrations from 10 to 2000 μg/ml were added directly to the microtubes. After the incubation at 37° C on a shaking device (120 r.p.m.) for 20 h, the microtubes were vortexed for 2 minutes to spread bacterial cells better in the supernatant. Then, the bacterial suspension was diluted serially, and 10 μl of each dilution was plated onto staphylococcus/pseudomonas isolation agar and was incubated overnight at 37° C. The viable bacteria in the biofilm were counted. To determine the remaining bacterial cells in the biofilms, treated biofilms were sonicated for 45s (NEXTGEN-CB17-LAB750, 40% Amplitude. 0.5 Cycle), serially diluted and subsequently plated onto isolation agar to determine colony counts. Counts were compared to CFU of PBS-treated control. The lowest concentration of the antibiotic required to cause a 99.9% reduction of CFUs with regard to that of the control growth was defined as the MBEC value [32, 38]. Antibiotic assay tests were conducted thrice in triplicate.

### MBEC determination of antibiotics in the presence of enzymes

Amikacin and meropenem with final concentrations through 10 to 1000 μg/ml were combined with the enzymatic mixture (50 U/ml DNase I**+**0.15 μg/ml trypsin) to evaluate how enzymes affect the susceptibility of *S*. *aureus-P*. *aeruginosa* biofilms to tested antibiotics. The MBEC value was measured after 20 h treatment as described previously.

### Statistical analyzes

Statistical analyses were performed in SPSS statistics software, version 24. Significant differences between the means of data were determined by Paired T-test and One-Way ANOVA. P-values *<* 0.05 were assumed statistical significance.

### Ethics statement

The study protocol was evaluated and approved by the Ethical Committee of Tehran University of Medical Sciences.

## Results

### Dual-species biofilm formation

**Fig 1:**
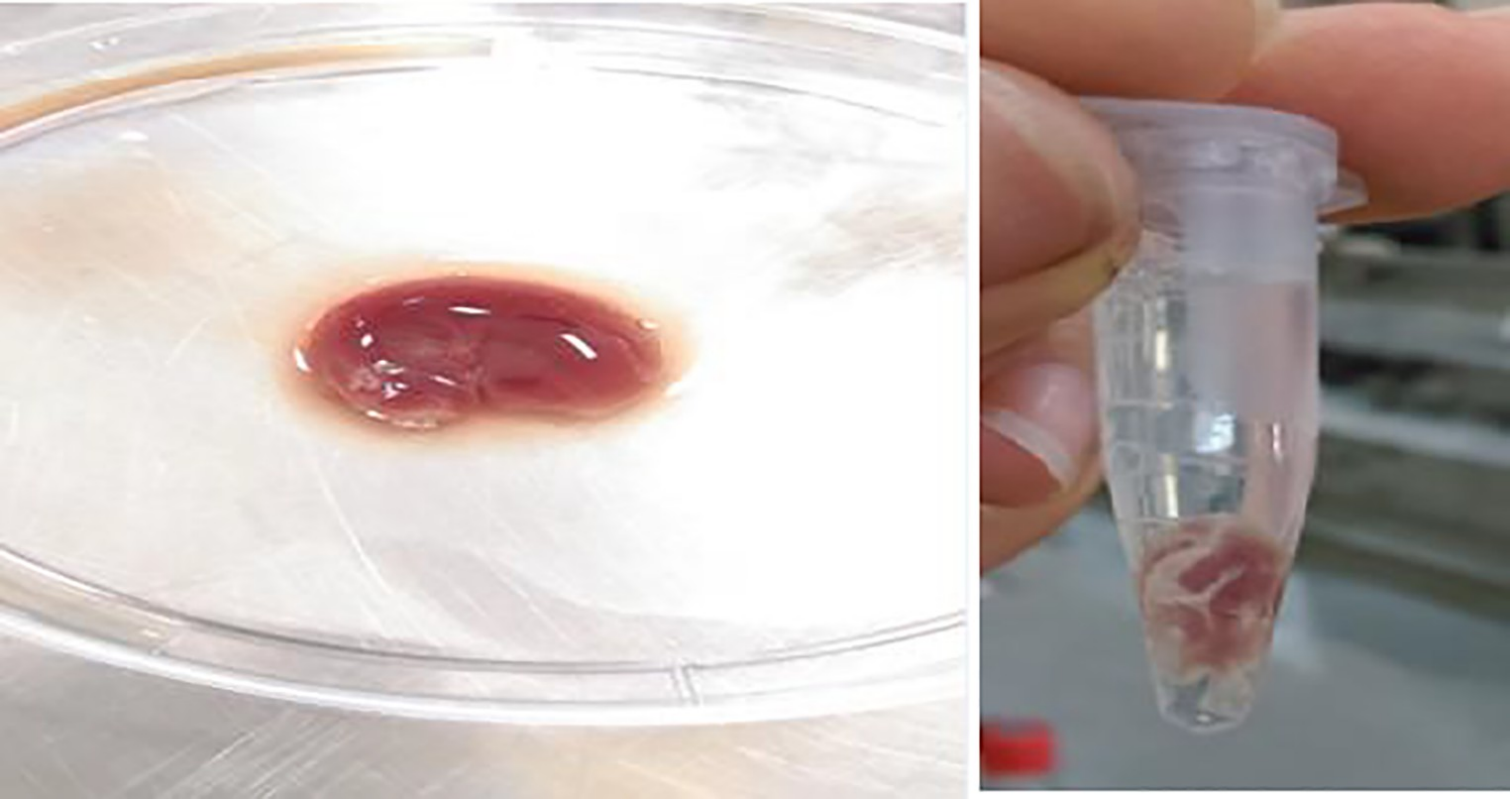
**Coagulated media in a plate (left) and in a microtube (right).** Wound-Like Medium containing heparinized human plasma, 45% Bolton broth base, 1% gelatin, and 5% laked sheep red blood cells, was inoculated with 10 μl of the combined and normalized culture (1×10^6^ CFU/ml) of *S*. *aureus-P*. *aeruginosa* bacteria. After 24 h incubation at 37° C, media was coagulated.

**Fig 2:**
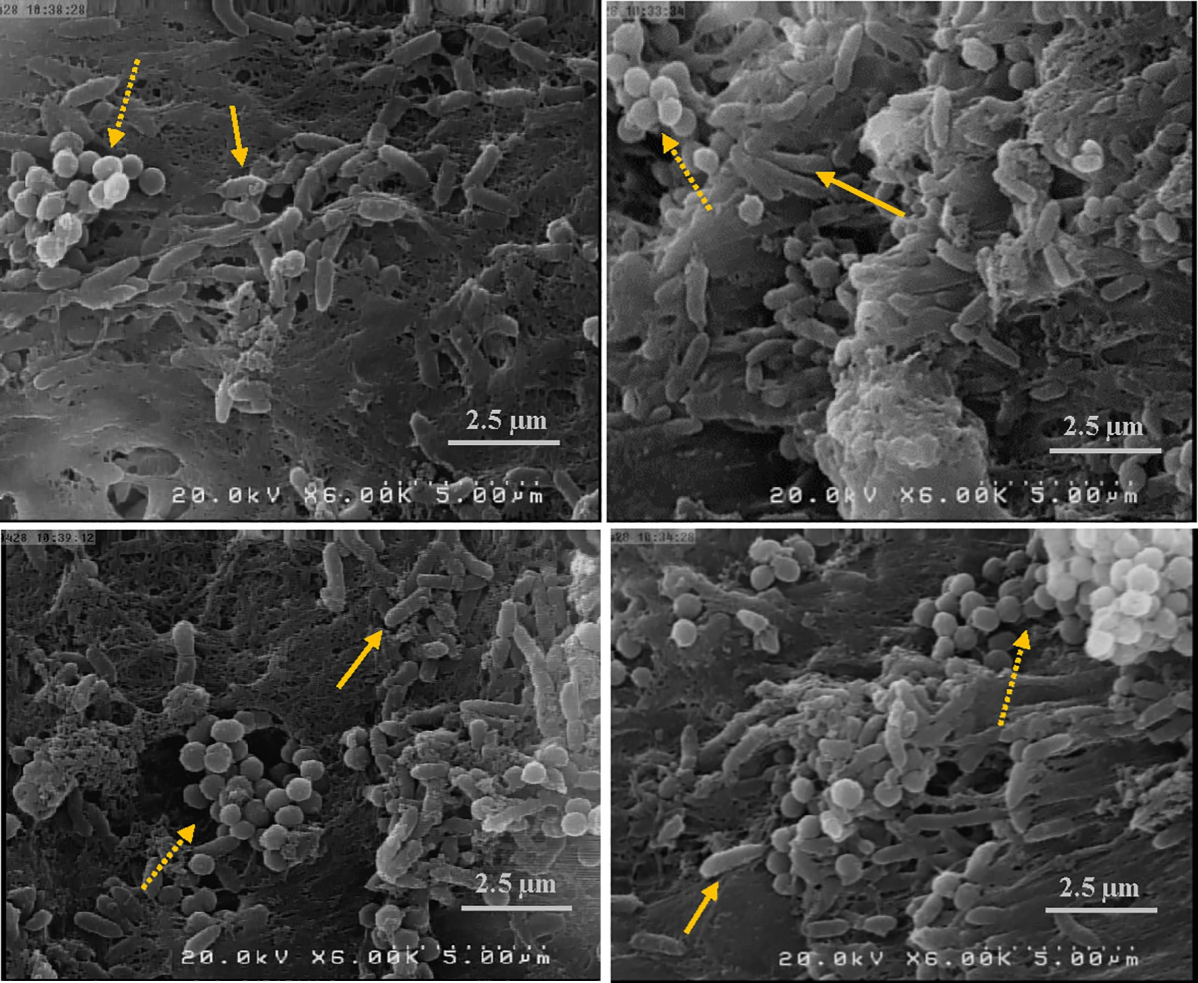
Scanning electron micrographs of *S*. *aureus-P*. *aeruginosa* biofilms. Rod (solid arrow) and cocci bacteria (dashed arrow) are shown. (6000x magnification).

After 24 h incubation of inoculated WLM at 37° C, the medium was coagulated (Fig 1). The coagulated media acts as a scaffold to biofilm formation. To ensure biofilm formation, bacteria were visualized in biofilms using SEM (Fig 2). Discrete clusters of rods and cocci could be seen in close proximity.

### The effect of enzymes on dual-species biofilms

The ability of trypsin, β-glucosidase, and DNase I enzymes to disrupt dual-species biofilms grown in WLM was tested. Preformed biofilms were treated with minimum effective concentrations of trypsin, β-glucosidase, and DNase I enzymes obtained 1 μg/ml, 8 U/ml, and 150 U/ml, respectively, for 18 h. It should be noticed that the enzymes at concentrations greater than their minimum effective concentration were capable of degrading biofilms at 4 or 8 h durations (S1 File). For instance, trypsin at 2 μg/ml and β-glucosidase at 16 U/ml degraded *S*. *aureus-P*. *aeruginosa* biofilm after 4 and 8 h, respectively. To avoid the probable toxic effect of the enzymes at high concentrations on animal model in future studies, we utilized the lowest effective concentration of tested enzymes. Biofilm-degrading effect of enzymes was determined by counting dispersed CFUs from biofilms into the supernatant. A significant increase in the number of bacterial cells dispersed in the environment was observed after treatment with tested enzymes but not with untreated control biofilms that had been buffer/PBS treated. It should also be noted that the overall number of CFU in supernatant plus biofilm in all treatment groups did not differ significantly and was approximately equal to total number of CFU in the supernatant of degraded biofilms after enzymatic treatment and dissolution of biofilms.

**Fig 3:**
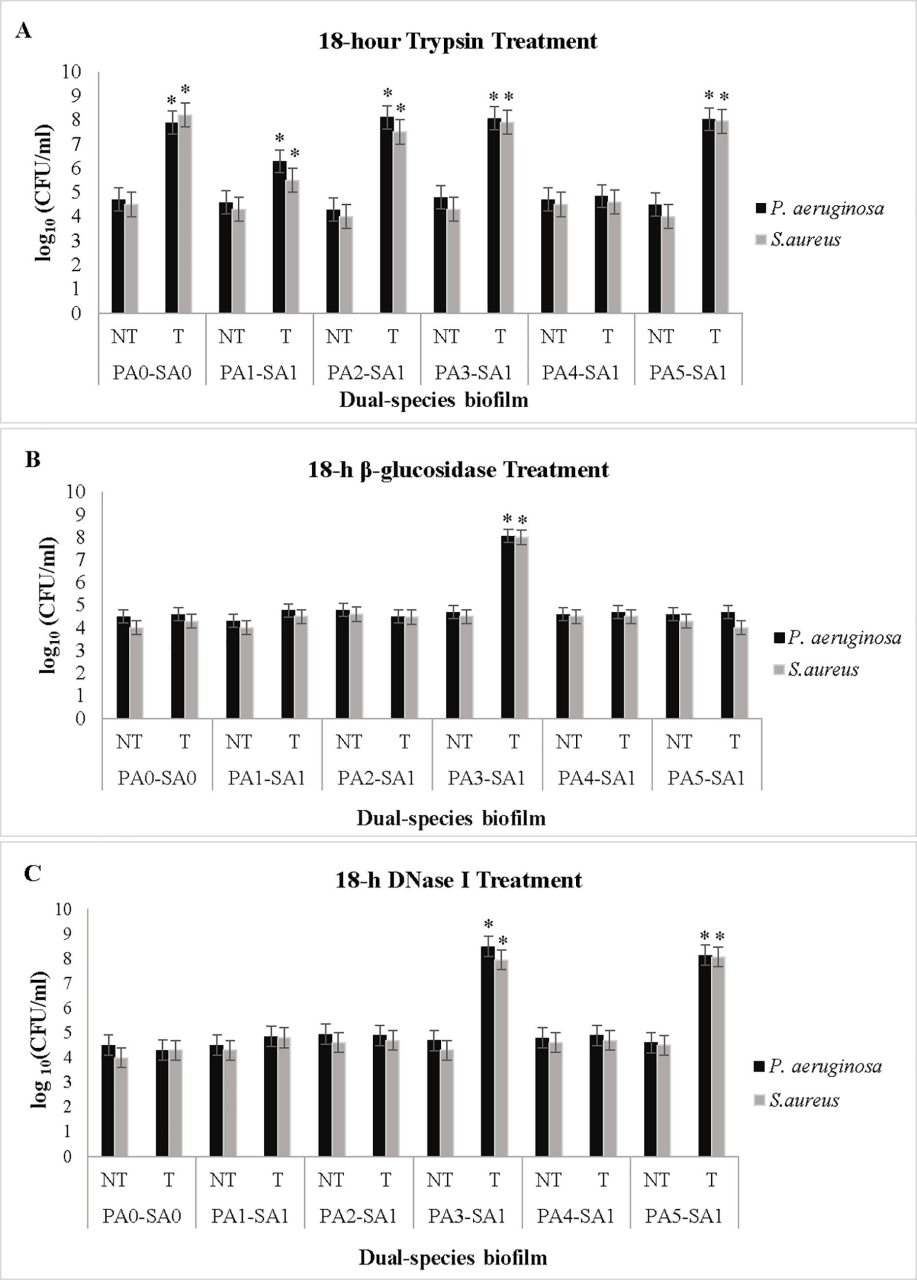
Dispersion of *S*. *aureus-P*. *aeruginosa* biofilms with 18 h enzymatic treatments. Biofilms developed in WLM and then treated with minimum effective concentrations of tested enzymes containing 1 μg/ml trypsin, 8 U/ml β-glucosidase, and 150 U/ml DNase I (T) or enzyme buffer (NT). After treatment, the number of dispersed bacterial cells were estimated by CFU enumeration on staphylococcus/pseudomonas isolation media. Graphs were drawn based on the logarithm of CFU/ml. Values represent the mean of three independent experiments with three replicates per condition. *Asterisks* indicate the statistically significant difference (*P< 0*.*05*) in the number of dispersed bacteria from treated biofilm compared to non-treated (control) biofilm. Error bars represent the standard errors of the means (SEM). Abbreviation: NT, Non-treated; T, Treated; PA_0_, PA ATCC 27853; PA_1_, PA1185; PA_2,_ PA1179; PA_3,_ PA1162; PA_4,_ PA1326; PA_5,_ PA1329; SA_0_, SA ATCC 29213; SA_1_, SA639.

Fig 3 shows the results of dispersal with enzymes at the minimum effective concentration on *S*. *aureus-P*. *aeruginosa* biofilms after 18 h incubation at 37° C.

Six series of *S*. *aureus-P*. *aeruginosa* biofilms were treated with trypsin enzyme with the concentration of 1 μg/ml, and trypsin reaction buffer was used as the test control (0 μg/ml). With the exception of PA_4_ (*pelF*^*-*^, *algD*^*-*^, *pslB*^*-*^, *pslD*^*-*^)-SA_1_ biofilm, trypsin caused a complete dispersion of four biofilm groups and partial degradation of PA_1_ (*pelF*^*+*^, *algD*^*+*^, *pslB*^*-*^, *pslD*^*-*^)-SA_1_ biofilm (Fig 3A). β-glucosidase at 8 U/ml concentration, just had significant (*p <* 0.05) dispersal effect on PA_3_ (*pelF*^*-*^, *algD*^*+*^, *pslB*^*+*^, *pslD*^*+*^)-SA_1_ biofilm (Fig 3B). DNase I at 150 U/ml concentration disrupted PA_5_ (*pelF*^*-*^, *algD*^*+*^, *pslB*^*-*^, *pslD*^*-*^)-SA_1_ and PA_3_ (*pelF*^*-*^, *algD*^*+*^, *pslB*^*+*^, *pslD*^*+*^)-SA_1_ biofilms (Fig 3C).

We found that trypsin/DNase I mixture was able to degrade *S*. *aureus-P*. *aeruginosa* dual-species biofilms. Due to the low pH of the β-glucosidase enzyme buffer (pH 5), its combination with other enzymes made them inactivated and had no degradative effect on biofilms.

**Fig 4:**
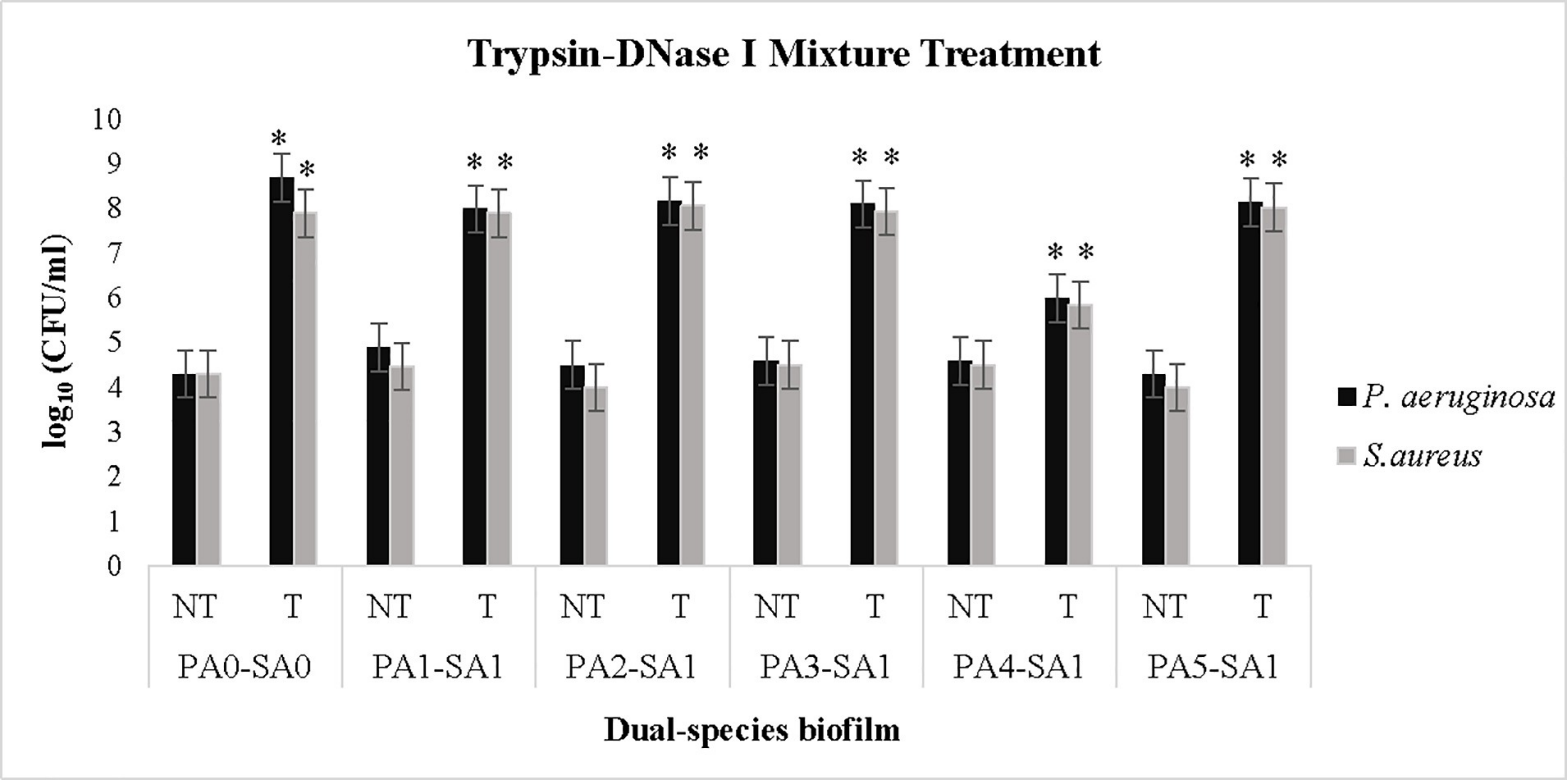
Effect of the trypsin/DNase I mixture on *S*. *aureus-P*. *aeruginosa* biofilms. Combination of 0.15 μg/ml trypsin and 50 U/ml DNase I enzymes was treated on established *S*. *aureus-P*. *aeruginosa* biofilms in WLM. The mixture degraded all biofilms completely and dispersed bacterial cells. Data show the logarithm of CFU/ml of biofilm-released cells. *Asterisks* indicate the statistically significant difference (*p <* 0.05) in the number of released bacteria from treated biofilms compared to non-treated (control) biofilms. The data shown are the mean (±standard error of the mean) of at least three replicates (three independent experiments). Abbreviation: NT, Non-treated; T, Treated; PA_0_, PA ATCC 27853; PA_1_, PA1185; PA_2,_ PA1179; PA_3,_ PA1162; PA_4,_ PA1326; PA_5,_ PA1329; SA_0_, SA ATCC 29213; SA_1_, SA639.

The results of combined enzyme treatments indicated that all trypsin/DNase I mixtures had a degradative effect on biofilms, except in the case of 0.15 μg/ml trypsin and 30 U/ml DNase I combination (S2 File). The combination of 0.15 μg/ml trypsin and 50 U/ml DNase I was considered as the minimum effective concentration in the mix and degraded all *S*. *aureus-P*. *aeruginosa* biofilms, causing the dissolution of biofilms and dispersal of cells into the environment (Fig 4).

Due to the dissolution of the susceptible biofilms to certain enzyme/enzymes (the remaining biofilm cells ~ 0), the log reductions corresponded to the results concerning the remaining cells within the biofilms are not presented.

### Bactericidal effect of enzymes on planktonic cells

To evaluate whether the enzymes have bactericidal effect, we determined MICs of each enzyme. Enzymes at their minimum effective concentrations and lower of which had no bactericidal effect (Table 3).

**Table 3:** MIC values of the enzymes against each strain.

	Strain
Enzyme PA_0_PA_1_ PA_2_ PA_3_ PA_4_ PA_5_ SA_0_ SA_1_	
**Trypsin (**μg/ml) >1 >1 >1 >1 >1 >1 >1 >1	
**DNase I (**U/ml) >150 >150 >150 >150 >150 >150 >150 >150	
**β-glucosidase** (U/ml) >8 >8 >8 >8 >8 >8 >8 >8	

Bactericidal effect of enzymes on each strain was performed using the minimum effective concentration of each enzyme and lower concentrations (Trypsin: ≤ 1 μg/ml, DNase I: ≤ 150 U/ml, β-glucosidase: ≤ 8 U/ml).

PA_0_, PA ATCC 27853; PA_1_, PA1185; PA_2,_ PA1179; PA_3,_ PA1162; PA_4,_ PA1326; PA_5,_ PA1329; SA_0_, SA ATCC 29213; SA_1_, SA639.

### Antibiotic susceptibility of isolates in different culture conditions

**Fig 5:**
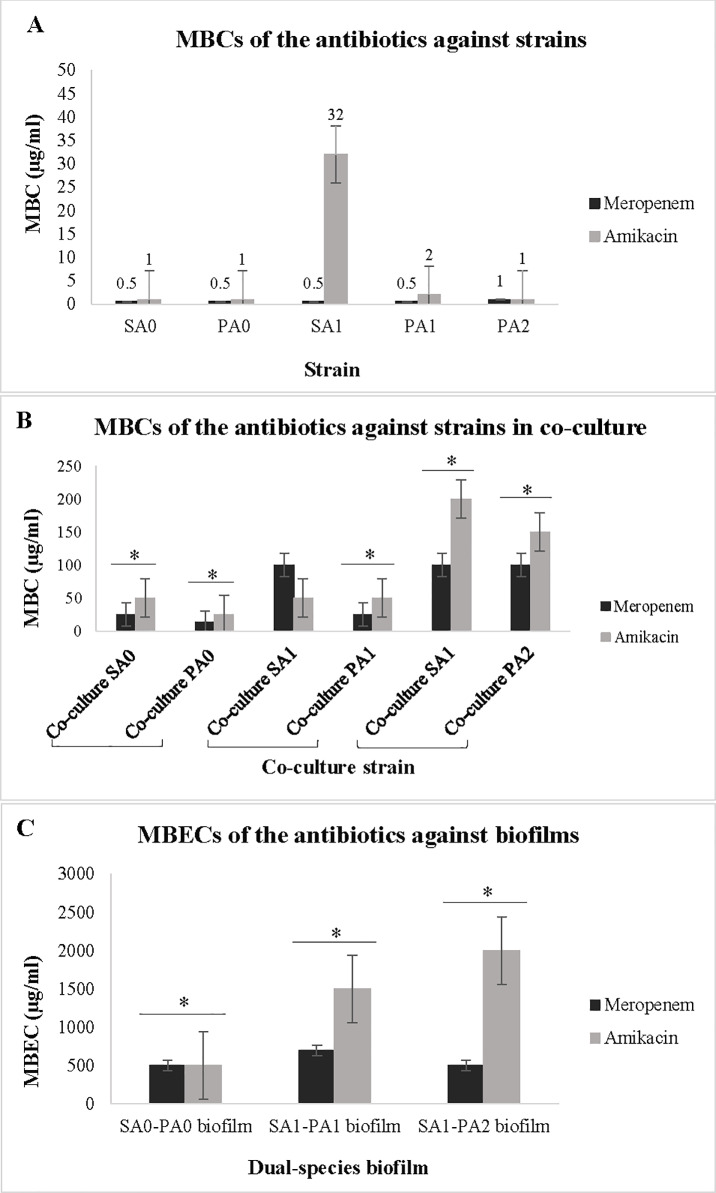
MBCs in different culture conditions. Data show the MBCs of meropenem and amikacin against monoculture strains, co-culture strains, and MBECs of the antibiotics against *S*. *aureus-P*. *aeruginosa* biofilms. A shows MBC values obtained against monocultures of SA_0_, PA_0_, SA_1_, PA_1_, and PA_2_ isolates. B indicates MBCs of the antibiotics against SA_0_, PA_0_, SA_1_, PA_1_, and PA_2_ strains when they were co-cultured (co-cultures: SA_0_-PA_0_, PA_1_-SA_1_, and PA_2_-SA_1_). C shows MBECs of the antibiotics against dual-species biofilms of SA_0_-PA_0_, PA_1_-SA_1_, and PA_2_-SA_1._ With the exception of SA_1_, all other strains showed a significant increase in MBC values in co-culture mode (B) over that of MBC values against monoculture strains (A). A significant increase in MBECs of meropenem and amikacin (C) compared to MBCs of antibiotics against co-culture (B) and monoculture (A) strains was observed. Values represent the antibiotic concentration in μg/ml. *Asterisks* indicate the statistically significant difference (*p <* 0.05) between MBC values in each chart (5B and 5C) compared to the previous chart/charts (5A and 5B). One-Way ANOVA was used to compare the MBC values obtained against monocultures, co-cultures, and MBECs (comparison among data of A, B, and C). The data shown are the mean of at least three replicates (three independent experiments).

As expected, isolates response to meropenem and amikacin was altered in different culture conditions. Isolates SA_0_, SA_1_, PA_0_, PA_1,_ and PA_2_ were susceptible to meropenem and amikacin in planktonic monocultures (Table 4). While, these susceptible strains showed high resistance to the antibiotics in co-culture and biofilm modes (Fig 5). Co-culturing *P*. *aeruginosa* and *S*. *aureus* altered their antibiotic susceptibilities and caused an increase of MBCs of the antibiotics against planktonic co-cultures (*p <* 0.05) (Fig 5B). Moreover, MBECs of meropenem and amikacin increased significantly compared to MBCs of antibiotics against co-culture and monoculture strains (Fig 5C).

**Table 4:** MIC results of the antibiotics against planktonic bacterial cells in monoculture.

Strain								
Antibiotic	PA_0_	PA_1_	PA_2_	PA_3_	PA_4_	PA_5_	SA_0_	SA_1_
**Meropenem**	0.25	0.5	1	128	64	128	≤ 0.25	0.25
**Amikacin**	1	2	1	64	512	128	1	16

Data indicate the antibiotic concentrations in μg/ml. MBC was determined for five susceptible strains including SA_0_, SA_1_, PA_0_, PA_1_, and PA_2_. The susceptible isolates were chosen to perform antibiotic assays for co-cultures and biofilms. Cut-off values defining susceptibility for meropenem and amikacin are ≤2 and ≤16, respectively (CLSI).

PA_0_, PA ATCC 27853; PA_1_, PA1185; PA_2,_ PA1179; PA_3,_ PA1162; PA_4,_ PA1326; PA_5,_ PA1329; SA_0_, SA ATCC 29213; SA_1_, SA639.

### MBEC reduction of meropenem and amikacin in combination with enzymes

**Fig 6:**
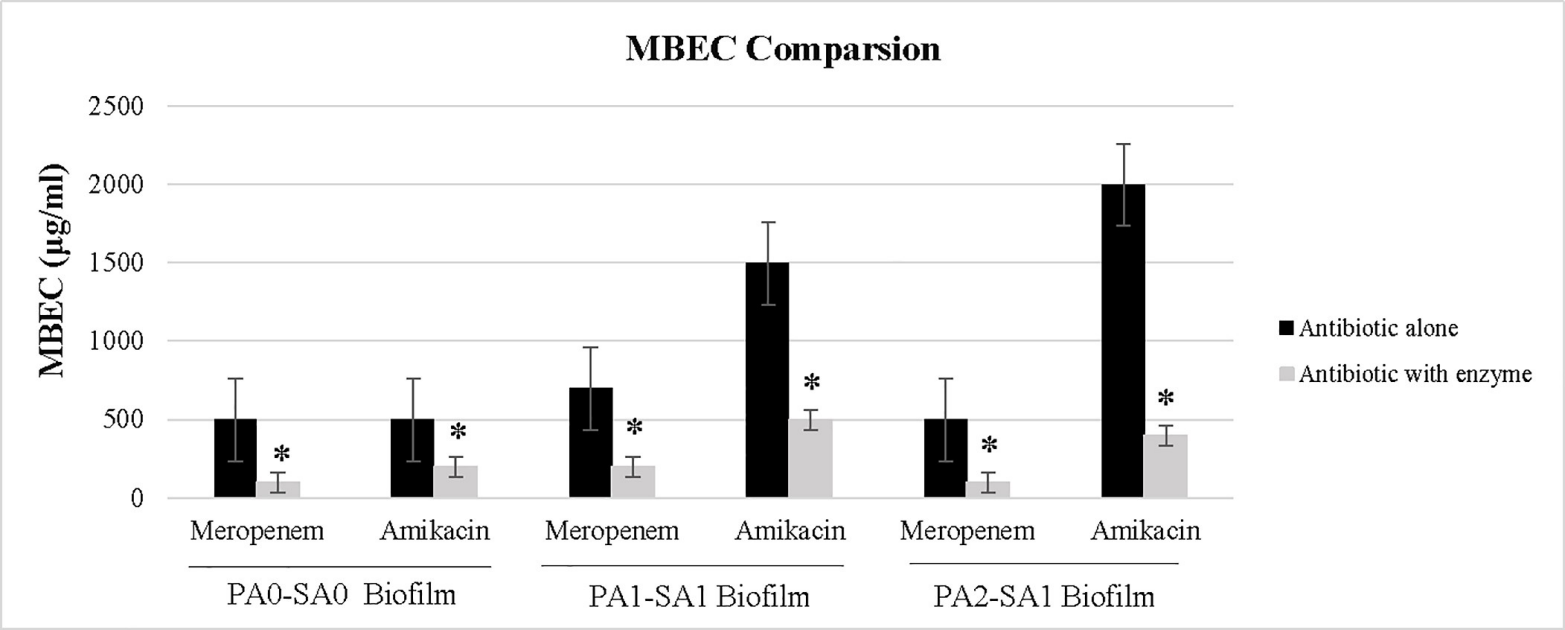
Comparison of MBEC results. Data shown represent MBECs of meropenem and amikacin alone (Antibiotic alone) and in combination with trypsin/DNase I mixture (Antibiotic with enzyme). MBECs of both antibiotics decreased significantly (*p <* 0.05) when combined with trypsin/DNase I mixture. Error bars represent the standard errors of the means.

Combination of enzymes with meropenem and amikacin decreased the MBECs significantly (*P<* 0.05) (Fig 6). Combination of the enzymatic mixture (0.15 μg/ml trypsin and 50 U/ml DNase I) with antibiotics achieved a reduction of 2.5 to 5 fold in MBEC of both meropenem and amikacin.

## Discussion

Biofilm formation is one of the most challenging issues in the antimicrobial therapy of infection, especially in chronic wounds, where multi-species biofilms display synergistic interactions. The interactions result in increased antibiotic resistance and hamper normal healing of wound [2, 5, 37, 39]. Finding alternative biofilm control strategies and eradication of biofilms in these wounds can be an efficient way to improve wound healing.

Numerous studies have shown the ability of different enzymes to disrupt biofilms *in vitro* and *in vivo* [10–16, 40–47]. In the current study, we investigated the effectiveness of trypsin, β-glucosidase, DNase I and combination of these enzymes on *S*. *aureus-P*. *aeruginosa* biofilms formed in WLM. The ability of the enzymes in the reduction of meropenem and amikacin MBECs was also evaluated.

The biofilm-degrading effect was observed in all of the examined enzymes but varied among the enzymes for the different biofilm groups of strains (Fig 3). This difference may be due to the variable composition of EPS between strains and due to different matrix composition in co-cultures resulting from interactions between two different bacterial strains.

Regarding the gene profiles of PA_1_, PA_4_, and PA_5_ strains, it seems that these strains lack Psl polysaccharide in their biofilm structures, which is one of the substrates of β-glucosidase enzyme. According to PA_2_ and PA_3_ genotypic patterns, it was expected β-glucosidase to be efficient on PA_2_-SA_1_ and PA_3_-SA_1_ biofilms. However, the β-glucosidase enzyme had no dispersal effect on PA_2_-SA_1_ biofilm. Probably, PA_2_-SA_1_ biofilm matrix contains fewer Psl polysaccharide content rather than other polysaccharides like alginate in its structure. It seems polysaccharides like Psl possessing target linkages of β-glucosidase may exist as essential components in PA_3_-SA_1_ biofilm matrix and could thus make the biofilm susceptible to this enzyme (Fig 3B).

Since only PA_3_-SA_1_ and PA_5_-SA_1_ biofilms responded to DNase I treatment, it could be suspected that PA_3_- SA_1_ and PA5—SA_1_ biofilms contain a larger proportion of eDNA in its matrix than the other isolates (Fig 3C).

Neither of the tested enzymes alone dispersed PA_4_-SA_1_ biofilm. Regarding the genotypic pattern of PA_4_ (*pelF*^*-*^, *algD*^*-*^, *pslB*^*-*^, *pslD*^*-*^), it seems other polysaccharides and/or proteins may be involved in biofilm matrix that are not accounted for in the genotyping. On the other hand, substrates of tested enzymes may exist in the biofilm structure at fewer amounts that their dispersion alone is not sufficient for dispersal of biofilm. Therefore, targeting these fragments by trypsin/DNase I mixture could make the biofilm susceptible to combination treatment and degraded PA_4_-SA_1_ biofilm (Fig 4).

The results (Fig 3) indicated that trypsin was more efficient on the biofilms than other tested enzymes. In concordance with our findings, some previous studies reported proteases as more effective enzymes than other depolymerizing enzymes [11, 14, 15]. Since proteins are one of the important components in the biofilm EPS, trypsin caused a massive dispersal event in dual-species biofilms. However, Banar et al. showed weaker effect of trypsin enzyme than mannosidase enzymes on *P*. *aeruginosa* mono-species biofilms since proteins are one of the sub components of *P*. *aeruginosa* biofilm [10, 48]. High efficiency of trypsin on *S*. *aureus-P*. *aeruginosa* biofilms in our study may be due to different EPS composition and vital structural role of proteins in dual-species *S*. *aureus-P*. *aeruginosa* biofilms.

Moreover, our data demonstrated that trypsin/DNase I combination led to a significant disruption of all *S*. *aureus-P*. *aeruginosa* biofilms of tested bacteria, while this effect was not observed using each enzyme alone, even at higher concentrations (Fig 4). This limitation can arise from the heterogeneity of the biofilm matrix [49]. The increased dispersion followed by the enzymatic treatment could be attributed to targeting two components of the biofilm matrix; eDNA and protein in biofilms concurrently. This observation indicates the mixture potential effect on *S*. *aureus-P*. *aeruginosa* biofilms in the clinical setting that can enable antibiotics and immune system to access dispersed, planktonic cells and contribute to an improved wound healing. Several previous reports suggest that the combination of biofilm-degrading enzymes would result in an improved dispersal of biofilms and antimicrobial efficacy of antibiotics in comparison with that obtained by the treatment of biofilms with the enzymes individually [50, 51]. Fleming et al. examined the efficacy of two glycoside hydrolases, α-amylase, and cellulase on *S*. *aureus-P*. *aeruginosa* biofilms grown in WLM and also *in vivo* [50]. Treatment of biofilms with these enzymes resulted in a significant biofilm degradation and an increase in the effectiveness of subsequent gentamicin treatments. Combination of these two glycoside hydrolases improved the efficacy of enzymatic therapy and led to a better biofilm dispersion. Tsiaprazi-Stamou et al. tested the efficacy of amylase, protease and lipase against a mixed microbial biofilm obtained from a meat packaging process line [51]. It was observed that the combination of enzymes was more efficient than formulations based in a single enzyme. The treatment with a formulation combining amylase, protease and lipase, effectively decreased the total biofilm mass. They found that despite lipids being present in a much lower amount than polysaccharides and proteins within the matrix, they might play a key structural role. In contrast, Waryah et al. demonstrated that the use of multiple biofilm-degrading enzymes in combination with each other may not necessarily result in a synergistic dispersal effect, but may reduce the overall antimicrobial efficacy of an antibiotic [44]. Their study revealed that both dispersin B (0.72 mg/ml) and DNase I (140 kU/ml) enzymes were equally efficient in enhancing the antibacterial efficiency of tobramycin (0.75 mg/ml) against S. aureus biofilm. However, a combination of these two biofilm-degrading enzymes was found to be significantly less effective in enhancing the antimicrobial efficacy of tobramycin than the individual application of the enzymes. These findings indicate that combinations of different biofilm-degrading enzymes may compromise the antimicrobial efficacy of antibiotics and need to be carefully assessed *in vitro* before being used for treating medical devices or in pharmaceutical formulations.

Since *S*. *aureus* and *P*. *aeruginosa* commonly coexist and are isolated from chronic wounds, we determined their susceptibility when grown in co-culture and dual-species biofilm. MBCs were assessed to evaluate MBEC reduction of antibiotics in combination with enzymes.

Because the biofilm matrix contributes to antimicrobial resistance, it would be expected that dispersed, planktonic cells resulting from enzyme degradative effect would be more susceptible to antibiotics. Despite high resistance in dual-species biofilms to both meropenem and amikacin, trypsin/DNase I mixture enhanced the activity of antibiotics against biofilms by disrupting and thus making them exposed and susceptible to antibiotics (Fig 6). These findings are in agreement with the previous reports describing the efficacy of biofilm-degrading enzymes on the enhancement of antibiotics/antimicrobials activity against bacterial biofilms [44, 46, 50, 52, 53, 54]. A study by Gawande et al. showed better antibiofilm-antimicrobial efficacy of Dispersin B^®^ and KSL-W combination against the chronic wound infection associated bacterial biofilm as compared to KSL-W peptide alone [46]. Dispersin B^®^ significantly enhanced the antimicrobial activity of KSL-W peptide against biofilm-embedded bacterial cells. The combination of Dispersin B^®^ (200 μg/ml) and KSL-W peptide (125 μg/ml) showed synergistic anti-biofilm and antimicrobial activity against chronic wound infection associated biofilm-embedded bacteria such as Methicillin-resistant *Staphylococcus aureus* (MRSA), *Staphylococcus epidermidis*, Coagulase-negative Staphylococci (CoNS), and *Acinetobacter baumannii*.

Saggu et al. used Peptidase M16 against S. aureus biofilm at 10 μg/ml, 100 μg/ml, and 1000 μg/ml concentrations [53]. They found that Peptidase M16 increased the penetration of kanamycin by degrading the bacterial EPS. The viability of bacterial cells in the biofilm decreased significantly on treatment with kanamycin (8x MBC) in the presence of protease (10 μg/ml). This effect was not observed when the biofilm was treated with kanamycin (8x MBC) or protease (10 μg/ml) alone. Recently, Trizna et al. showed that extracellular levanase SacC from Bacillus subtilis (at 1 mg/ml) disrupts the matrix biofilm of *P*. *aeruginosa* and increases the efficacy of ciprofloxacin and amikacin antibiotics against biofilm-embedded bacteria *in vitro* [54].

According to the results, MBECs in the presence of enzymes were higher than MBCs against co-cultures, which can be resulted from high tolerance of biofilm-resident cells. Likewise, antibiotic resistance increased significantly within *S*. *aureus-P*. *aeruginosa* co-cultures compared to monocultures that can be explained by synergistic interactions between species (Fig 5). Similarly, the previous study by DeLeon et al. demonstrated an increase in tested antibiotics tolerance levels in co-cultures of *S*. *aureus-P*. *aeruginosa* over that of monoculture cells [37]. Moreover, another study by Dalton et al. indicated that the bacteria in the multispecies wound infections displayed increased antimicrobial tolerance in comparison to those in mono-species infections. It seems synergistic interactions between different bacterial species may contribute to antibiotic tolerance [55].

Our findings suggest that trypsin/DNase I mixture could be used as an agent to remove *S*. *aureus-P*. *aeruginosa* biofilms. The use of an agent that would disperse the biofilm could allow the appropriate antibiotic to act upon the infection would improve the chronic wound healing. Disruption of biofilm could thus help to avoid the debridement of wounds and result in less pain in patients [56]. It also can lead to a shorter stay in the hospital and a reduction of health-associated costs.

The current study encounters several limitations. Firstly, while the trypsin/DNase I enzymatic mixture exhibited promising results against dual-species *S*. *aureus-P*. *aeruginosa* biofilms, its efficiency against biofilms in chronic wounds remains questionable. Therefore, we consider retesting the enzymatic efficacy *in vivo* in an animal model in future studies. Moreover, toxicity assays with the enzymes could be useful to investigate cytotoxic effects of the enzymes on skin cells. Another limitation is related to the selection of strains. Selecting different *S*. *aureus* strains with variety in genes encoding EPS components would result in a more accurate interpretation of the effect of enzymes on biofilm groups.

## Conclusion

In conclusion, it was illustrated that the combination of trypsin**/**DNase I enzymes targeting different components of biofilm matrix, can be considered as an anti-biofilm agent and an appropriate candidate to degrade *S*. *aureus-P*. *aeruginosa* biofilms. It could also have potential applications for degradation of biofilms on medical devices and different surfaces in medical care units to prevent nosocomial infections, even if it may not be utilized in an *in vivo* application. Presumably, the combination of a polysaccharidase with trypsin/DNase I mixture in a similar buffer condition, may improve and broaden enzymatic mixture efficacy on a wide range of biofilms and introduce a novel high-potential agent to fight against a variety of biofilms.

## Supporting information

S1 File This file contains the data used to determine the minimum effective concentration and the best contact duration time for each enzyme. It also contains the data and results concerning enzymatic treatment of the biofilms with different concentrations of each tested enzyme at various contact duration time.
(XLSX)

S2 File This file contains the information representing the degradative effect of different trypsin/DNase I mixtures on dual-species biofilms. (XLSX)
